# Retrocolic Fascia—An Anatomical and Multidetector Computed Tomographic Angiography (MDCTA) Morphometric Analysis in Patients with Right Colon Cancer

**DOI:** 10.3390/diagnostics14171952

**Published:** 2024-09-03

**Authors:** Antoine Chemtob, Dejan Ignjatovic, Bojan V. Stimec

**Affiliations:** 1 Anatomy Sector, Teaching Unit, Faculty of Medicine, University of Geneva, 1211 Geneva, Switzerland; antoine.chemtob@etu.unige.ch; 2 Department of Digestive Surgery, Akershus University Hospital, University of Oslo, 1478 Lorenskog, Norway; dejan.ignjatovic@medisin.uio.no; 3 Institute of Clinical Medicine, University of Oslo, 0372 Oslo, Norway

**Keywords:** Gerota’s fascia, anatomy, MDCTA, CME, right colon, cancer

## Abstract

Background: This study aims to delineate anatomical landmarks crucial for complete mesocolic excision, focusing on Gerota’s fascia, which guides surgical dissection in right-sided colon cancer, forming the posterior limit. Employing a multimodal approach, the research aims to understand the fascial anatomy and its variations under pathological conditions. Methods: Three methods were applied: a pilot dissection on an embalmed cadaver for clear anatomical presentation of prerenal fascia, Mimics segmentation of the fascia and its relationship with the colon, and a retrospective analysis of MDCTA scans from 196 patients (mean age 65.73 y, 118 F/78 M). Systematic measurements of fascial thickness were taken at key renal levels—upper pole, hilum, lower pole, and infra-renal. Covariates analyzed included Body Mass Index, age, and sex. Results: The pilot dissection revealed the renal fascia of Gerota as the only true retrocolic compact connective tissue and the fusion fascia of Toldt as a mesh of strands of loose connective tissue and fat lobules. MDCTA showed clearer visualization of Gerota’s fascia at the hilum and inferior renal pole, predominantly on the left. There were significant differences in fascial thickness between sides (1.30 mm on the right and 1.34 mm on the left) and a positive correlation with BMI, whereas age and sex showed no significant effects. Conclusion: Gerota’s fascia is a critical anatomical landmark in CME for right colon cancer. This study highlights the fascia’s structural integrity, unaffected by the tumor, underscoring its importance in surgical navigation.

## 1. Introduction

Fasciae represent crucial landmarks in many surgeries, such as colectomy, nephrectomy, duodenal pancreatectomy, etc. They define precise anatomical spaces and determine dissection planes. These structures are defined by Wendell-Smith [1] as webs, sheets, or other dissectible aggregates of connective tissue visible to the naked eye. However, a clear definition supported by precise descriptions and iconographic documentation is needed [2]. This article will discuss retroperitoneal fasciae.

The parietal peritoneal fascia represents the connection between the parietal peritoneum and the ascending/descending mesocolon and is better known as the Fascia of Toldt, corresponding to the visceral plane during complete mesocolic excision (CME) [3].

Posteriorly, the anterior renal fascia, also known as Gerota’s fascia, corresponds to the anterior part of the aponeurotic envelope surrounding the adipose renal capsule. Cranially, Fredet’s fascia, also called anterior pancreatic fascia [4], constitutes the attachment between the mesoduodenum/pancreas (visceral duodenal–pancreatic peritoneum) and the mesocolon [5]. On the same somatotopic plane posteriorly, Treitz’s fascia [6], or retro-duodenal-pancreatic fascia [7], links the mesoduodenum/pancreas to the parietal peritoneum.

These four fasciae are important landmarks in abdominal surgery. Knowledge and identification of these different structures constitute the essential basis for colon surgery, particularly during extended mesenterectomy for cancer, first described by West [8] in 2008 and Hohenberger [3] in 2009.

Total mesorectal excision (TME) used in the treatment of rectal cancer was the inspiration for the extended mesenterectomy approach. However, CME also involves ligation of the colic vessels at the level of their origin (CVL) and extensive lymphadenectomy [9], comparable to the D3 dissection previously described.

The first step of CME of the hepatic angle and transverse colon was described by Açar et al. [10]. The root of the superior mesentery is mobilized using the medial-to-lateral approach [11], penetrating the embryological avascular plane and surgically separating the fascia covering the posterior mesocolon from the parietal fascia (Gerota’s fascia), which, in turn, covers the anterior part of the retroperitoneum. Continued dissection is accomplished by keeping a parietal anchorage on the lateral edge of the colon at the level of the Toldt white line [12] and then proceeding in a cephalic direction. Finally, after complete mobilization of the right and transverse colon, the central vascular ligation can be performed.

The dissection plane to mobilize the ascending, descending, and sigmoid colon lies within the Toldt’s fascial layer [13]. It is crucial, however, to keep the mesocolon intact during CME to reduce the risk of tumoral leakage [7,14].

Because understanding the posterior limit of the CME approach is key to the surgery, this study will focus on this parietal plane represented by Gerota’s fascia. As all the patients referred for colectomy undergo a CT scan, an exact preoperative CT assessment of the retrocolic fasciae can be of importance for the correct complete mesocolic excision technique, leading to an appropriate oncological surgery.

This study will be achieved through a multimodal approach, involving firstly the presentation of a pilot dissection of the region of interest, and secondly, a multidetector computed tomographic angiography (MDCTA) analysis of the prerenal fascia. This imaging will provide a technical description of the fascia through systematic measurements and will detect the potential impact that right-sided colon cancer might have on its thickness. One of the salient points is whether the fascia is homogenous throughout, or whether there might be some weak zones that can endanger proper surgical procedure. Measurements of the fascia thickness are to be made bilaterally at different levels on preoperative CT axial slices in patients suffering from right colon cancer, with BMI, sex, and age used as covariates. The analyses of the left-sided prerenal fascia will provide a case-matched control for the right side.

## 2. Materials and Methods

The core of this study was preceded by two pilot studies: dissection and image reconstruction. The first was performed on an embalmed human body (male, age 85 years) from the body donor program of the Anatomy Sector, Faculty of Medicine, University of Geneva, Switzerland. The use of the human cadaveric material was performed according to the Federal Act on Research involving Human Beings (Human Research Act, HRA) [15], the Guidelines of the Swiss Academy of Medical Sciences [16], and the principles of the Swiss Society of Anatomy, Histology, and Embryology (SSAHE) [17]. Donors officially agreed to the use of body parts for research purposes by personally signing the body donation statement form [18]. The exclusion criteria for this portion of the study were chronic or acute diseases of the digestive tract, past abdominal surgery, and infectious diseases.

The body was injected with a Jores solution, consisting of 1875 mL 40% formaldehyde, 750 mL chloralhydrate, 750 g Carlsbad salt, and 12,375 mL distilled water and then kept in a vacuum plastic bag at 5 degrees celsius. After conditioning to the ambient temperature, the minute dissection began with a wide semicircular incision of the anterolateral abdominal wall, enabling access to the infracolic compartment of the peritoneal cavity. The greater omentum was retracted upward, the right colic angle pulled gently to the left, and the peritoneum was shallowly incised along the right paracolic gutter. The right colic angle, ascending colon, the cecum, and their corresponding vessels were gradually pulled by the aid of an Israel retractor to the left, and the right retrocolic space was dissected in a stratigraphic manner, identifying layer by layer. The subserosal fatty tissue was removed by gentle scraping, using narrow medical spatulas, microdissection scissors, small tweezers, and curved forceps. On approaching the macroscopically dissectible blade of dense connective tissue, the surrounding loose connective and adipose tissues were cleaned off. This compact fascia was incised vertically, and deeper contents were observed. Throughout the dissection, the working field was regularly sprayed, i.e., kept humid by a phenol solution, preserving the original consistency.

The pilot image reconstruction, as well as the main trunk of the study, was carried out by AC and BVS on the datasets from the clinical trial ‘Safe Radical D3 Right Hemicolectomy for Cancer through Preoperative Biphasic Multidetector Computed Tomography Angiography’, which received the IRB approval REK Sør-Øst No. 2010/3354, Norway, and was recorded at https://clinicaltrials.gov/ (NCT01351714) [19]. This trial includes patients with a histologically proven adenocarcinoma of the right colon, planned for elective surgery and under clear guidance by the inclusion criteria (age under 75 years, cleared for general anesthesia, informed consent signed) and exclusion criteria (recurrent disease, distant metastases, or peritoneal dissemination). A prerequisite for entering this study was a preoperative computerized multidetector computed tomography angiography (MDCTA) of the abdomen and pelvis, with a millimetric or submillimetric slice thickness. In the case of the pilot study, the dataset was with double contrast colography (air and barium sulfate). It was analyzed by manual segmentation using the Mimics medical image processing software, ver. 24.0, and 3-matic medical software, ver. 16.0 (both for Windows 10 Pro x64 (Materialise NV, Leuven, Belgium). The DICOM dataset was imported into a blank Mimics project and then underwent profile line manual thresholding in order to obtain sufficient voxel value for the aimed structures. Two initial reconstructions of the large bowel were carried out, one with air as a negative contrast and the other with barium as a positive contrast. These two 3D masks were attributed the same color and then fused, giving the full contour of the colon. Further, the initial mask was cropped and underwent detailed and minute single- and multiple-slice editing with automated interpolation, tracing, and marking of the extension of the Gerota’s fascia related to the colon 3D mask. Manual editing was facilitated by Dynamic region growing, Split mask, 3D LiveWire, Morphology operations, and Boolean operation tools. Finally, a joint 3D object mask of the colon and retrocolic fascia underwent the “Calculate Part” option without any postediting, such as smoothing or triangle reduction, so that the original form is preserved. The calculated colon–fascia mask was fused and exported as still images, STL, and video files.

**Figure 1:**
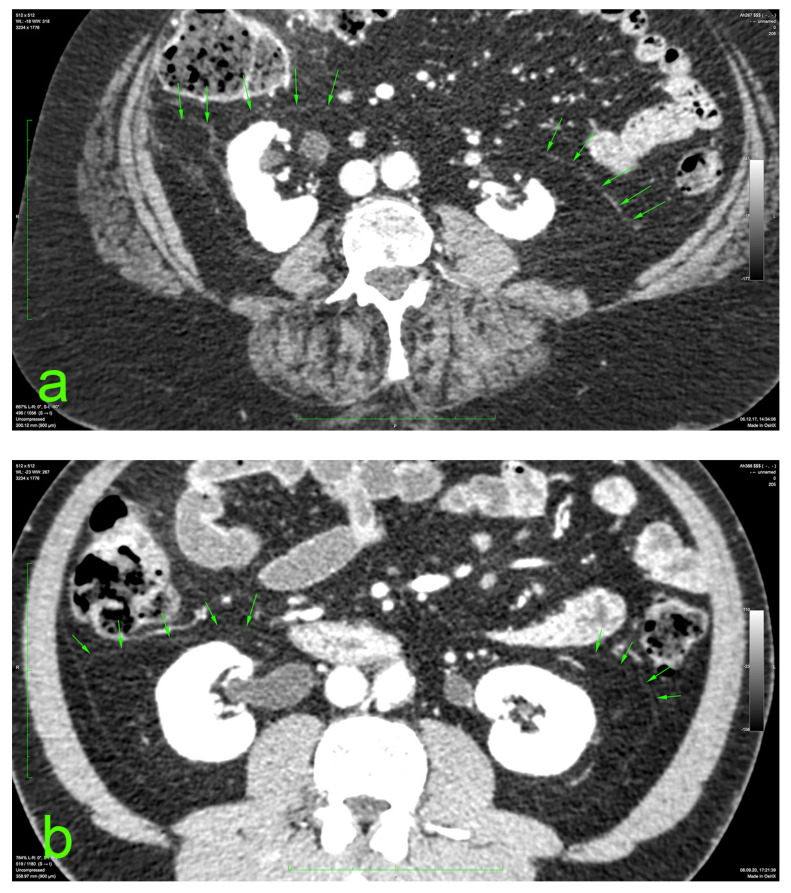
Prerenal fasciae (green arrows) on injected CT: (**a**) 70-year-old female patient with a BMI of 32.9; (**b**) 50-year-old male patient with a BMI of 30.1.

The principal portion of this study was also based on the aforementioned CT datasets. They were analyzed using a 2D multiplanar reconstruction with a maximum intensity projection with the aid of Food and Drug Administration (FDA)—approved Osirix MD v.14.0 64-bit image-processing application software (Pixmeo, Bernex, Switzerland). The wide area behind the ascending and descending colon was analyzed for the appearance of the distinct retrocolic fascia. The thickness of this fascia was measured at four arbitrary levels, related to the kidneys, on both sides (left and right): upper pole, midlevel, lower pole, and below the lower kidney pole. Each measurement was performed twice, calculating the arithmetic mean for each level. The final thickness (tool Length) of one side was defined as the mean value of the four measurements (Figure 1).

The statistical analysis was conducted using Visual Studio Code 1.86.2 (Universal), Electron 27.2.3, ElectronBuildId 26908389, Chromium 118.0.5993.159, Node.js 18.17.1, V8 11.8.172.18-electron.0, on Darwin arm64 23.1.0 operating system, with Python [3.11.5] (2024), including the Power Analysis, Sample Size Calculation, Kolmogorov–Smirnov test for normality of distribution, and comparisons between values measured on the right and the left made using the Wilcoxon–Mann–Whitney test. The technique of least squares was applied through Ordinary Least Squares (OLS) regression to assess the relationship between the CT scan measurements and the independent variables: Body Mass Index (BMI), age, and sex. Pearson correlation coefficient for continuous variables was used, and Spearman rank order correlation or Fisher exact test was employed for categorical variables. In order to investigate the specific influence of BMI on the right and left thickness, we used Spearman’s rank correlation test. The significance was set at a *p*-value of 0.05.

## 3. Results

### 3.1. Pilot Study

The pilot dissection revealed that the only true retrocolic compact connective tissue formation is the renal fascia of Gerota. The fusion fascia of Toldt is a mesh of strands of loose connective tissue and fat lobules in the vicinity of the posterior colic wall and that are adherent to it and contain the paracolic and epicolic blood and lymph vessels.

**Figure 2:**
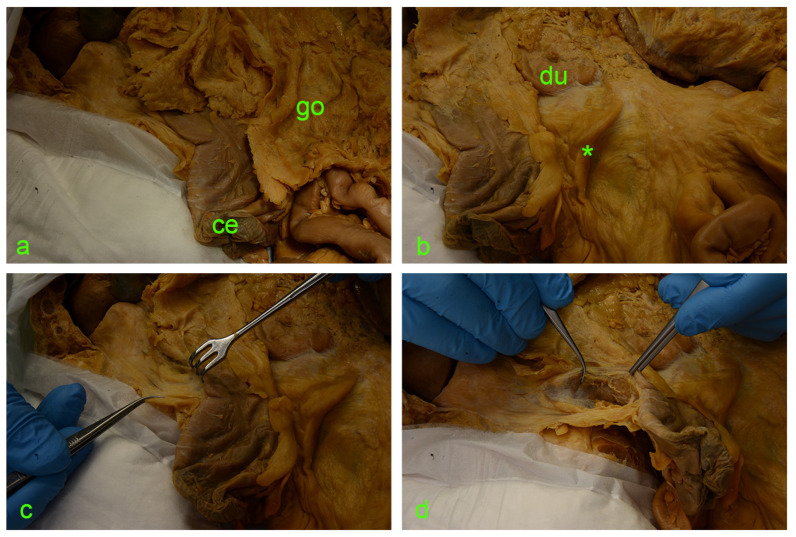
(**a**) ce—cecum; go—greater omentum covering the intestinal loops. (**b**) Asterisk—ileocecal fold; du—duodenum. (**c**) Israel retractor pulling the ascending colon medially, exposing the paracolic peritoneal fusion line (a.k.a. white line of Toldt). (**d**) Incision of the peritoneum along the white line of Toldt and exposure of the retrocolic space.

**Figure 3:**
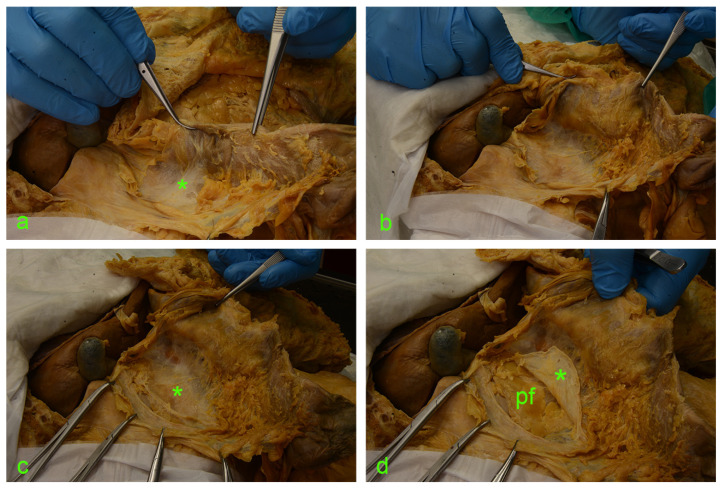
(**a**) asterisk—fusion fascia of Toldt with a ‘hair-like’ aspect. (**b**) fusion fascia of Toldt pulled along with the ascending mesocolon to the left. (**c**) Full exposure of the right retrocolic space; under the fusion fascia lies the anterior renal fascia (asterisk). (**d**) A vertical incision was made along the the Gerota’s fascia (asterisk); cutting through this connective tissue envelope allows visualization of the perirenal adipose tissue (pf).

The following images (Figure 2 and Figure 3) illustrate the step-by-step sequential dissection of the mentioned area.

### 3.2. MDCTA

**Figure 4:**
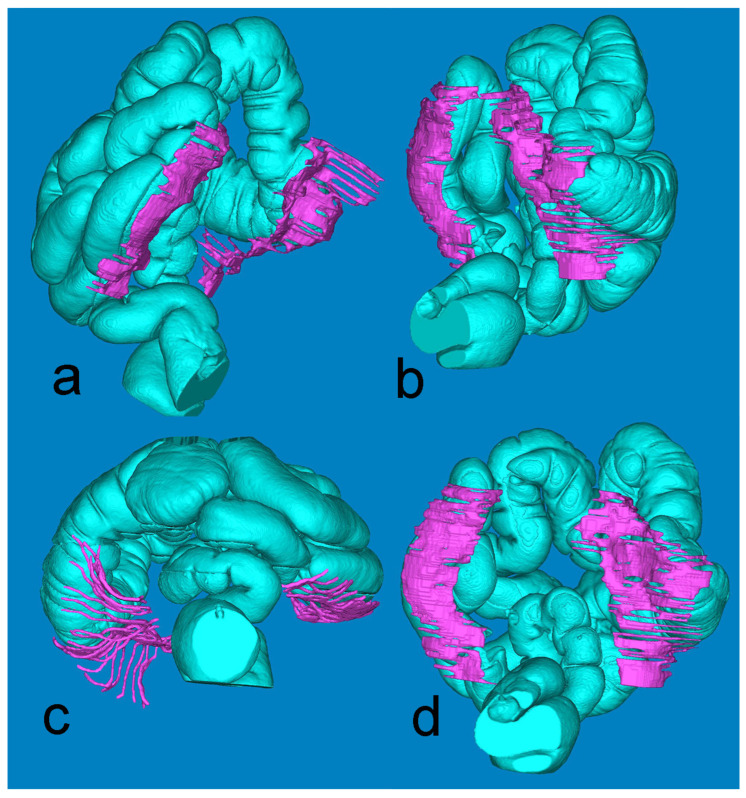
Mimics reconstruction of the large bowel (turquoise) and the adjacent retrocolic fascia (fuchsia). Views: (**a**) left oblique; (**b**) right oblique; (**c**) axial, from below; (**d**) coronal, posterior.

Figure 4, derived from the minute Mimics-based segmentation, gives a synoptic view of the extension of the retrocolic fascia bilaterally. The full presentation of the large intestine is achieved by fusing the air-based and barium-based 3D models.

### 3.3. Statistical Analyses

The group of patients selected for this study comprised 196 individuals (118 women—60% and 78 men—40%). The average age of the participants in our study was 66 years. The mean BMI value was 27.1 ± 5.2 (min–max: 17.1–45.5). The results of the thickness measurements are presented in Table 1 below.

**Figure 5:**
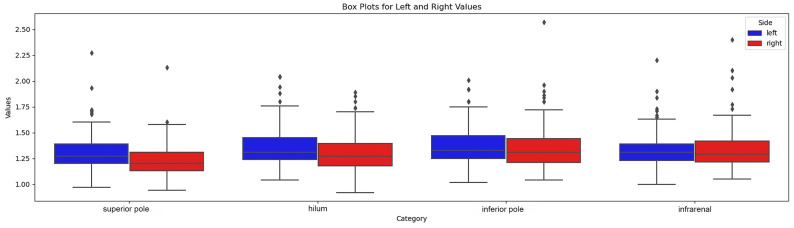
Box-plot of fascial thickness at 4 levels.

Overall, the visibility of the anterior renal fascia was slightly better on the left side, as reported in the visibility grid (Table 2). We found that the most cranial plane was the level where the fascia was least visible, on both sides. The fascia in the infra-renal plane was also more difficult to visualize. On the other hand, sections at the hilar level and inferior renal pole provided the best visibility of the fascia. The anterior renal fascia was better visualized in men (7% missing values) than in women (10% missing values), although, in both sexes, we still had poorer visibility in the most cranial plane (superior renal pole) and more generally on the right. The variant fascia thicknesses at predefined levels are given in Figure 5.

We first assessed the distribution of the data we took from the CT images using the Kolmogorov–Smirnov normality test.

This showed that only the values measured on the right-hand side at the kidney upper pole and hilar level follow a normal distribution according to this test.

We therefore compared the mean measurements taken on the right side with the mean of those on the left using the Wilcoxon–Mann–Whitney test. A statistically significant difference was noted between the distributions of measured values on these two sides.

As shown in the summary table, the left anterior renal fascia is slightly thicker than that of the contralateral side.

**Figure 6:**
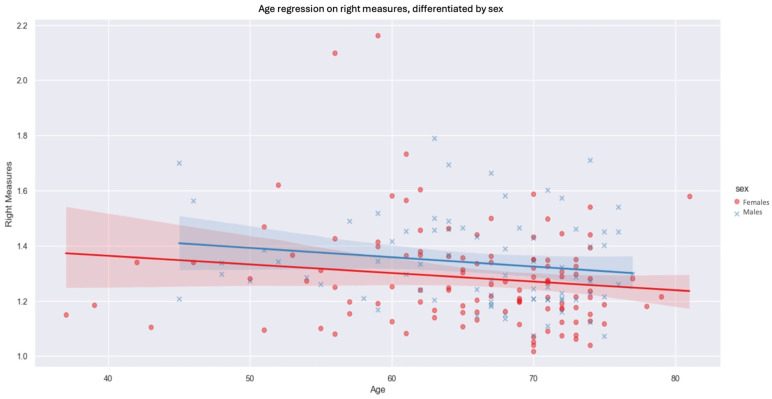
Age regression on right measures, differentiated by sex (blue xs, lines and shadows = males, red dots, lines and shadows = females; line—mean, shadow—SD.

We then observed the influence of sex and age on the thicknesses measured on each side, keeping the four unilateral levels average. On both sides, men had increased fascia thickness compared with women by 0.0501 mm on the left (*p*-value: 0.036) and 0.0556 mm on the right (*p*-value: 0.031). The negative inclination of the regression suggests that age has an inverse influence on fascia measurements (Figure 6). Indeed, as patient age rises, we observe a downward trend in the values measured. The influence of age is slightly greater on the right side than on the left. In contrast, for the left side, the influence of age on prerenal fascia thickness was not statistically significant at our threshold of *p* < 0.05.

However, by adding BMI as another comparison parameter, we observe that the influence of sex and age on measurements is no longer statistically significant.

**Figure 7:**
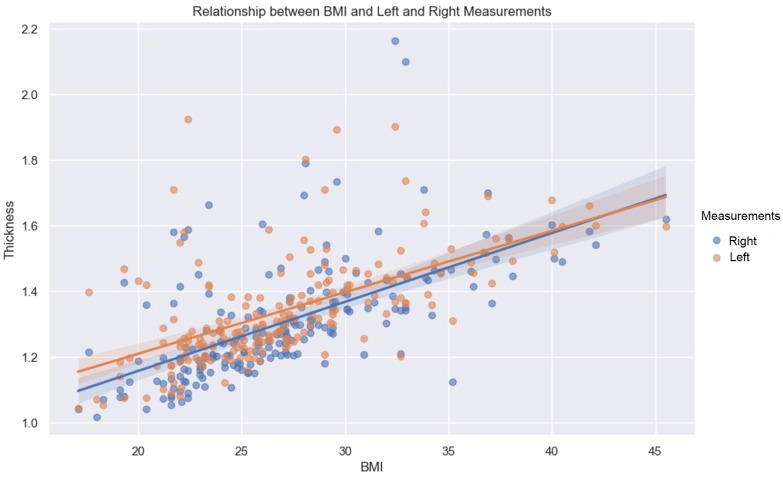
Relationship between BMI and left (orange dots) and right (blue dots) measurements. Line—mean, shadow—SD.

BMI has a constructive influence on measurements (Figure 7). This strong and statistically significant association is demonstrated by a Spearman correlation of 0.66 (*p*-value < 0.05, IC95: [0.57, 0.73]) for the mean of the values measured on the left, and a Spearman correlation of 0.7 (*p*-value < 0.05, IC95: [0.62, 0.76]) on the right.

As demonstrated by this correlation, the association with BMI is slightly greater on the right than on the left, for both sexes.

**Figure 8:**
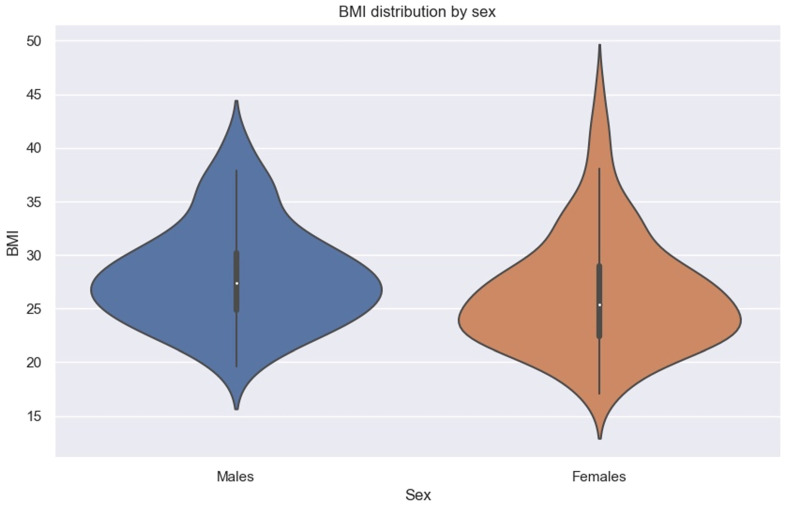
Violin plot showing BMI distribution by sex.

We must specify that in our sample, men had higher BMIs than women (Figure 8).

**Figure 9:**
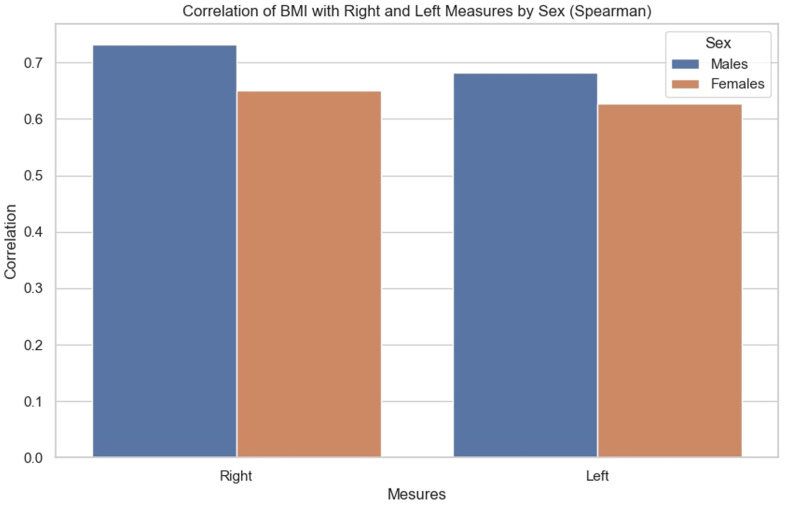
Bar chart showing correlation of BMI with right and left measurements by sex (Spearman correlation).

The correlations were then compared. We sought to observe for each BMI the influence it had on the measurements of each side, as well as whether there were any observable differences between men and women. It was noted (Figure 9) that the correlation between BMI and anterior renal fascia thickness is slightly greater in men than in women.

## 4. Discussion

Improved survival rates in patients with right colon cancer have been made possible by the introduction of a complete excision technique [20]. This is based on dissection within the embryonic planes and is known as CME with CVL [3,8]. To carry out this dissection according to this embryological plane, it was necessary to describe it clearly. The first description of this zone was given by Toldt, who gave his name to the fusion fascia found there. The anatomist proposed the embryological hypothesis that this fascia arose from the fusion of the visceral peritoneum of the colon and its mesentery with the parietal peritoneum of the retroperitoneum. He proposed that during this process, the epithelial layer would be lost, and the underlying connective tissue fused to create this Toldt fascia. It is now accepted that the visceral mesenteric layer and the parietal-peritoneal layer are maintained intact into adulthood and that the Toldt fascia develops between these two layers.

In the present study, the interest of the multimodal approach was to offer a better understanding of the ‘retrocolic’ fascia, as well as an analysis of a posterior component, Gerota’s fascia. This entity corresponds to the retromesenteric and combined interfascial planes of the newly introduced concept of the interfascial spread [21]. The retromesenteric plane divides the pararenal and the perirenal space; apart from it, there is also the retrorenal, lateroconal, and combined interfascial plane. The latter is a fusion of the retromesenteric and retrorenal planes and lies below the inferior pole of the kidney.

CT identification and analysis of the prerenal fascia can be challenging at certain places, which was noted mainly at the level of the upper renal pole and particularly on the right, as formerly shown by Frezza et al. [22]. We suppose that this could be due to its proximity to the liver on this side, as the different connective envelopes can become indistinct on imaging. Visualization of the fascia in the infra-renal plane was challenging, particularly due to the considerable anatomical variation in its attachments to the anterior surface of the quadratus lumborum [23].

Furthermore, in people with a low BMI and therefore a reduced amount of adipose tissue in the peri- and para-renal spaces, visibility of the fascia was also more difficult, which is in line with the same concept previously mentioned concerning proximity to solid organs. The ‘partial volume’ effect is greater on the fascia due to its oblique path (Figure 4), which may also be a cause of difficulty in visualizing the prerenal fascia. All these obstacles exist even though CT device technology has advanced considerably, and all CTs included in this study had millimetric or submillimetric slice thickness.

Subsequently, by comparing the measurements between the two sides (left–right), we were able to observe that the fascia on the left side was thicker than that on the right. This result came as a surprise, given that all the patients included in the study had noninvasive right-sided colon cancer. Indeed, we expected to observe a potential sclerosing or irritant effect from this cancer given the fascia’s anatomical proximity to the colon. We anticipated that this would manifest itself as a thickening of the right anterior renal fascia.

The result obtained suggests several hypotheses. The first would be that other, more anterior structures, would receive the contingent changes inflicted by the cancer. As presented in the histopathological study of Shan Wang et al. [14], the mesocolon is not only the envelope containing the lymph nodes, nerves, fat tissue, and vascular supply system but also a barrier to tumor cells. We could therefore assume that this layer would be the main target of tumor influences.

In addition, Gerota’s fascia is a thick and less elastic fibrous sheet as compared with other thin, elastic visceral fasciae, which are closely connected to the organs and give them shape, supporting the parenchyma [24] (lung fascia, liver fascia, and abdominal visceral fasciae, for instance). These histological differences between the fasciae are further arguments in support of the difference in influence of the cancer, albeit at some length, on the thickness of the prerenal fascia.

It has been shown that connective tissue elements and epithelial cancer have a complex biochemical interplay [25]. This would tend to reduce the thickness of the fasciae. To substantiate this hypothesis, it would be necessary to compare the measurements taken on these patients with right-sided colon cancer with other CT measurements taken on healthy people or on people with left-sided colon cancer.

One of the main findings of this study is that Gerota’s fascia is uneven in thickness. This would have some value in the clinical setting, since falling out of the correct layer would be easier in some areas than in others (e.g., right versus left and other places). An operating surgeon could possibly inform himself on the location of these ‘weak’ areas from the preoperative staging CT and, in this manner, evade injury to the fascia.

When measuring Gerota’s fascia, we subsequently investigated the influence of sex, age, and BMI on fascia thickness. In our results, we observed that BMI has a progressive correlation and statistically significant influence on fascia thickness. However, for sex and age, we were unable to prove such an influence.

This observation initially points to the need to extend the application of the measurements to a larger sample of patients to determine their impact. It is also possible that these two parameters have no effect on the anatomical structure concerned. However, at this stage of our research, it is premature to draw any definitive conclusions.

As highlighted for the first time by Frezza et al. [22], prerenal fascia visibility is more demanding in people with a low BMI. It is therefore important to highlight a potential measurement and comparison bias in this population compared with people with higher BMIs. This will remain the case until fascia detection and measurement capabilities are not improved for patients with low amounts of retroperitoneal adipose tissue.

Finally, the patients included in the study all had right colon cancer, but we were not interested in other pathologies that could affect them. Given the absence of thickening on the right side compared with the left, it is wise to consider other interferences.

Although one of the criteria in selecting patients for the study was the radiological absence of renal pathology, the influence of processes occurring within the perirenal space, which could affect the thickness of the fascia, must also be considered. For instance, on CT imaging of patients with locally advanced renal cell carcinoma (renal cell carcinoma pT stage > T3a according to AJCC, 7th edition, 2010), thickening of Gerota’s fascia appears to be a significant sign of involvement, with a 90% specificity and an 82% PPV, according to Bradley et al. [26].

The main strength of this study is that it addresses a relevant research question regarding different anatomical landmarks used for the correct complete mesocolic excision technique. The limitation of this study is that the CT analysis was based on a dataset with right colon cancer. It would be interesting to compare our findings with those without visceral pathology, if they are possible to obtain.

## 5. Conclusions

In this study, we, as well as other authors, succeeded in visualizing the prerenal fascia as a distinct anatomical entity, thus confirming its importance in retroperitoneal anatomical architecture. CT visualization of this fascia proved to be of good quality, enabling accurate and reliable identification. Notably, we observed that the thickness of Gerota’s fascia correlates with BMI, a finding that clearly stands out on CT imaging. However, our study failed to establish any significant correlation regarding the influence of right colon cancer, age, or sex on prerenal fascia characteristics.

In complete mesenteric excision surgery (CME) for right colon cancer, precise visualization of the prerenal fascia is essential. The prerenal fascia serves as a posterior boundary, marking the target area while also marking the limit that must not be overpassed. This delineation is even more relevant in patients with a low BMI, where visualization of the fascia is more difficult. Thus, our results underline the importance of careful assessment of the prerenal fascia in the preparation and performance of CME, offering a pathway to safer and more precise interventions.

## Figures and Tables

**Table 1:** Imaging measurements of Gerota’s fascia thickness (left, right, and total).

	Right Side (mm)	Left Side (mm)	Mean Thickness (mm)
Mean ± SD	1.3 ± 0.18	1.34 ± 0.16	1.32 ± 0.16
Min	1.02	1.04	1.04
Max	2.16	1.92	2.03

**Table 2:** Visibility grid of Gerota’s fascia in relation to the corresponding kidney.

	Visibility	
Level	Right Side	Left Side
Upper pole	69%	78%
Hilum	93%	99%
Lower pole	97%	98%
Infra-renal	92%	87%

## Data Availability

The data presented in this study are available on reasonable request from the corresponding author due to the IRB decision of the study and the protection of the anonymity of the participants. The data are available only for the purpose of scientific evaluation.
